# Special Issue “Interplay between Fungal Pathogens and Harvested Crops and Fruits”

**DOI:** 10.3390/microorganisms9030553

**Published:** 2021-03-08

**Authors:** Dov B. Prusky, Edward Sionov

**Affiliations:** 1 Department of Postharvest Sciences, Institute of Postharvest and Food Sciences, Agricultural Research Organization—The Volcani Center, Rishon LeZion 7505101, Israel; 2 Department of Food Sciences, Institute of Postharvest and Food Sciences, Agricultural Research Organization—The Volcani Center, Rishon LeZion 7505101, Israel; edwardsio@volcani.agri.gov.il

**Keywords:** fruit pathogenicity, postharvest pathogens, postharvest losses, *Penicillium*, *Botrytis*, *Colletotrichum*, toxins

## Abstract

The interplay between fungal pathogens and harvest crops is important in determining the extent of food losses following the storage and transport of crops to consumers. The specific factors modulating the activation of colonization are of key importance to determining the initiation of fungal colonization and host losses. It is clear nowadays from the wide number of transcription studies in colonized fruits that pathogenicity in postharvest produce is not only the result of activation of fungal pathogenicity factors but is significantly contributed to fruit maturity and ripening. In this editorial summary of the Special Issue “Interplay between Fungal Pathogens and Harvested Crops and Fruits”, we present a short summary of future research directions on the importance of the interplay between fruit and pathogens and nine published papers (one review and eight original research papers), covering a wide range of subjects within the mechanism of pathogenicity by postharvest pathogens, including transcriptome analysis of pathogenesis, pathogenicity factors, new antifungal compounds and food toxin occurrence by pathogens. This summary may lead the reader to understand the key factors modulating pathogenicity in fruits.

Colonization of postharvest diseases during storage and shelf life is the result of significant metabolic and physiological changes occurring in the fruit that enables the activation of quiescent and wound-penetrating pathogens. The specific factors modulating the activation of colonization are the result of a wide range of host and pathogen interactions modulating the final colonization development and pathogenicity. It is clear nowadays from the wide number of transcription studies in colonized fruits that pathogenicity in postharvest produce is not only the result of activation of fungal pathogenicity factors but is significantly contributed to by biochemical changes occurring during host maturity and ripening [1,2,3]. We believe that stage-specific interactions contribute to the process of fungal penetration and colonization, resulting in decay development. 

Pathogens can penetrate postharvest crops directly through wounds during the harvest period and storage handling, or through the cuticle in preharvested commodities. In both cases, disease symptoms are strongly dependent on the etiology of the fungal attack. A wide group of host factors may determine the initial interaction that determines the penetration of the pathogen into the host. These include the release of volatile mono terpenes from wounded citrus fruits that inhibit or enhance germination and penetration of the postharvest pathogen in to the citrus host. The germination and appressoria formation by *Colletotrichum musae*, for example, may be induced by the specific release of fruit phenolic leachates in banana that were uptaken and metabolized by the pathogen. Appressoria was also induced by specific cutin epicuticular waxes present in the avocado fruit cuticle that enhanced appressoria formation by *Colletotrichum gloeosporioides*. Other specific host morphological structures, such as cuticle thickness, may determine the capability of germinated appressoria to colonize the fruit by modulating a rapid or slower penetration of *Monilinia* to nectarines. The presence of high concentrations of phenolic compounds in the intracuticular wax cuticle of peaches and mandarins inhibited cutinolytic enzymes, produced by the germinating appressoria of *Monilinia* or by germination of *Penicillium* spores, which are needed for them to establish in peaches or mandarin fruits. More recently, specific host cuticle morphology was reported to determine the germination and penetration of *Botrytis* to grapes. In this case, fruit susceptibility was determined by the thickness and hydrophobicity of the wax layer that enabled, in non-hydrophobic wax surfaces, fluent nutrient transport from the fruit-enabled *Botrytis* germination and grape susceptibility. These examples indicate that specific initial interactions may determine the penetration and final colonization of the host.

However, while the interplay between the pre-infection and penetration processes showed a number of specific interactions, the process of quiescence after penetration, concluding in the active postharvest development of pathogens, is far from showing options that are clearly understood [4]. Quiescence in host fruits is an extended sequence of time during which pathogen ingress appears to be suspended. Owing to its prolonged nature and the dependency of developmental cues on fruit maturity, postharvest pathogens that have reached the internal milieu of the host develop new approaches to be ready to reinitiate their development during the active stage. Alkan et al. [5] suggested the expression of 178 genes that could be defined as quiescent-specific in *Colletotrichum*. Cell cycle components were upregulated, including *Cgl-Pps1* phosphatase, which has a role in DNA synthesis and in gene encoding for cyclin which is essential for control of the cell cycle. Histone modifiers and ATP-dependent chromatin remodeling complexes regulating DNA accessibility that are crucial for gene activation/repression were upregulated specifically at the quiescent stage. This includes genes related to histone deacetylase, histone-lysine-methyltransferase and epigenetic silencing via heterochromatin assembly that replace modified acetyl groups with methyl groups. Additionally, *RNA polymerase II mediator subunit 21* was upregulated 100-fold. All this indicates that the interplay between the host and pathogen quiescence, both histone modifiers and modification of the chromatin structure is strongly activated, probably building up the basis for the pathogen’s followed attack by upregulating genes, contributing to pathogenicity. This is one subject that needs further study in the future. One of those genes is the activation of glutamate dehydrogenase crucial for ammonia production during necrotrophic colonization, which is needed for creating the alkaline environment required for *Colletotrichum* necrotrophic development.

During this quiescent interplay, the host transcripts are also highly upregulated. As described in several cases of transcriptomic analysis, a prominent source of fruit metabolic energy remained enhanced by fatty acid degradation [1,2,6,7]. Defense pathways that were activated included the phenypropanoid pathway for phytoalexin and lignin precursors, such as cinnamic, cumarayl, coniferyl, caffeoyl, shikimic, quinic and sinapyl derivatives. Relevant transcripts for the synthesis of phenylpropanoid derivatives as potential substrates for peroxidase activity are also increased in expression to prevent the oxidation of phenols that limit fungal growth. Taken together, the results implying induction of antifungal compounds, phytoalexin, biosynthesis and lignification comprise a major ongoing fruit defense pathway employed by the fruit in response to persistent presence at initial stages of quiescence. In avocado fruits, upregulation of fatty acid metabolism was detected during the quiescent interplay of avocado and *Colletotrichum*. *C. gloeosporioides* inoculation of unripe fruits induced activation of membrane nicotinamide adenine dinucleotide phosphate (NADPH) signaling and enhancement of Δ^9^stearoyl-acyl-carrier protein (ACP) desaturase activity, resulting in the accumulation of unsaturated 18:2 products, which are basic precursors for preformed and inducible antifungal dienes [4]. 

Hormonal upregulation of ethylene production is known to commence during the later ripening stage of fruit ripening. However, during the quiescent interplay, the ethylene pathway signaling components are usually reported to be upregulated [5]. This includes the upregulation of transcripts for ripening-related, ethylene-sensitive, transcription factors NOR (non-ripening), RIN (ripening inhibitor) and CNR (colorless non-ripening). Furthermore, ethylene-regulated defense genes that are usually massively expressed include *class 1 chitinases*, *pathogenesis-related protein 1* and *pathogenesis-related protein 10*. These results suggest that while quiescent fungi stimulate fruit ethylene production and defensive responses, they will also enhance the ripening process and hasten the release from quiescence. Another hormonal mechanism activated during the quiescence interplay is the jasmonate pathway. Jasmonic acid and ethylene are known to co-activate defense reactions in a synergistic fashion, while ABA (abscisic acid) is antagonistic to their action, raising the question as to why all three pathways appear to be co-activated at the transcriptional level. 

In unripe tomato, the quiescent interplay includes activation of the mevalonate pathway and accompanying vetispiradiene synthesis in response to appressoria, probably towards producing the main sesquiterpenoid phytoalexin of tomato fruit, rishitin. Transcriptome revealed significant concomitant upregulation of key steroid glycoalkaloid (e.g., tomatine) transcripts (glycoalkaloid metabolism) *GAME4* and *GAME7*. The *GAME7* homolog represents the first committed step of the pathway that converts cholesterol to glycoalkaloid, while *GAME4* controls the metabolic junction that converts saponin-aglicones to steroidal-alkaloids such as α-tomatine [8]. Significantly, abundance of *GAME* transcripts decrease drastically during later fruit ripening, which would compromise biosynthesis of steroidal-alkaloids. This supports the hypothesis that ripe fruits have reduced amounts of antifungal compounds, which permit emergence of fungi from quiescence [4]. All broad host and fungal activities during the quiescent period of development indicates that both the host and the pathogen showed specific metabolic profiles that contribute to activation of the colonization process. 

Following the quiescent interplay, the activation of pathogenicity genes is the key factor contributing to the decay pattern. Postharvest pathogens have evolved the ability to respond and adapt to environmental pH changes after harvest in ripening fruits [4]. Ambient pH usually acts as an important signal for a large number of cellular events, including growth, morphogenesis, membrane and cell wall stability, protein stability and function, secondary metabolism and host infection. Contribution of pH changes during the transfer from quiescent to active infection is widely present in postharvest pathogen colonization [4]. For the optimization of fungal colonization, fruit pathogens usually secrete pH-modulating molecules, identified as organic acids or ammonia, which acidify or alkalize the host environment during the initial stages of necrotrophic colonization in ripe fruit. *Penicillium expansum* and *Penicillium digitatum* can acidify their host’s tissue by secreting gluconic acid and *B. cinerea* and *Sclerotinia sclerotiorum* by secreting oxalic acid, thereby activating several polygalacturonases that contribute to pectin depolymerization and thus tissue maceration [4,9]. Furthermore, the pathogen can secrete significant levels of ammonia at the leading edge of the colonized tissue, temporally modulating the activation of PacC [10]. 

During the activation of the quiescent infection and following the ammonification of the tissue, the functional form of PacC present in alkaline conditions is translocated into the nucleus, where it regulates the expression of PacC-dependent genes. These genes are involved in numerous biological functions, including growth, secondary metabolism and virulence in several fungal pathogens of humans, plants and insects and also regulating the virulence of a number of pathogenic fungi. Deletion of PacC homologs reduced fungal virulence in plant pathogens *S. sclerotiorum*, *Colletotrichum acutatum*, *C. gloeosporioides*, *Penicillium digitatum* and *P. expansum* [10,11]. Transcriptome analysis of pH control in several postharvest pathogens, such as *P. expansum* and *C. gloeosporioides*, revealed an arsenal of pathogenicity factors, transporters and antioxidants to control virulence and homeostasis under changing ambient pH conditions. Comparison of diverse fungal genomes showed a similar strategy of control by PacC, indicating a conserved role in regulating fungal genes. The differential pH regulation of genes with similar activities suggests that they are selectively activated based on their optimal enzymatic pH activity, allowing the fungus to cope with variable pH conditions and make optimal use of the available enzymes. The results indicate that several PacC-regulated genes have a major impact on pathogenicity following the activation of the quiescent infections, which is considered to be the main regulator of pathogenicity in postharvest pathogens. 

The Special Issue “Interplay between Fungal Pathogens and Harvested Crops and Fruits” enabled the publication of a significant series of articles, which focus on physiological, biochemical and molecular aspects of host–pathogen interactions. The work of Zhang and co-workers [12] described a new essential oil cuminal obtained from *Cuminum cyminum* that was proven to control growth and colonization of the postharvest pathogen *Trichothecium*. Transcriptomic analysis of the pathogen treated by cuminal showed that the affected systems by the oil are located in the membrane system and cytosol of the fungus, along with ribosomes, mitochondria and peroxisomes. Enrichment analysis showed that lipids and amino acid degradation, ATP-binding cassette transporters, membrane reconstitution, mRNA surveillance pathway and peroxisome were upregulated, whereas secondary metabolite biosynthesis, cell cycle and glycolysis/gluconeogenesis were downregulated. Zhang and co-workers concluded that the essential oil mechanism of action activated oxidative stress and damaged organelles, resulting in energy deficiency to fungal growth, contributing to fungal starvation and inhibition of fungal growth.

Three different papers concentrated on the mechanism of pathogenicity of *Penicillium* sp. in citrus and apple hosts. The work of Qian et al. [3] on the “Elucidation of the initial growth process and the infection mechanism of *Penicillium digitatum* on postharvest citrus (*Citrus reticulata* Blanco)” attempts to identify the early changes that contribute to symptom development. They described *P. digitatum* as showing very rapid germination in the first hours of infection accompanied by the accumulation of organic acid and soluble sugars from dead cells at the infection point. The genes related to cell wall degrading enzymes were significantly upregulated. The transcriptomic profile of genes 44 h after inoculation showed early expression of PG1 compared to lower expression of cellulases, suggesting a differential timing of expression for both enzymes during pathogenicity. They concluded that organic acid(s) accumulation and PG1 expression are the initial factors modulating the very early infection process of *P. digitatum* in wounded tissues. This study provides good information on the infection mechanism of *P. digitatum* on postharvest citrus during the initial infection process.

A similar concept in “Comparative transcriptomic analysis of the interaction between *Penicillium expansum* and apple fruit during early stages of infection” was explored by Wang et al. [2]. They identified an early interaction in *P. expansum* that was related to cell wall degradation enzymes, antioxidative stress, pH regulation and effectors. A total of 3, 13 and 32 upregulated CWDEs were identified at 1, 3 and 6 h after inoculation, respectively. The antioxidative genes were already detected as far as 3 h after infection, suggesting a very rapid response to host ROS production. Relative expression of the glucose oxidase genes increased by 8-fold on the 6 h postinoculation, suggesting that pH regulation occurs during fungal germination and before symptom occurrence. Interestingly, apple tissues responded to the presence of *P. expansum* by activating pathogen-associated molecular pattern (PAMP)-triggered immunity at 1 h after infection, then activated effector-triggered immunity at 3 h postinoculation. This indicates a balance between the mechanisms of pathogenicity activated by the pathogen and the host response at very early stages of fungal penetration of the apple tissue and, at the same time, a faster response of the interaction than that reported by Qian et al. [3] during the penetration of *P. digitatum* into the citrus host. These very early results indicate that while no quiescent infections were defined in wound-penetrating *P. expansum* to apples, significant interplay between the host and the pathogen is observed at this early stage of colonization.

The third manuscript of this series deals with pathogenicity factors from *Penicillium digitatum* by Ballester et al. [13]. The authors analyzed the “Functional and pharmacological analyses of the role of *Penicillium digitatum* proteases on virulence”. Their approach was to study the role of *P. digitatum’s* proteases in virulence by deletion and functional analysis of the *prtT* gene, which codes for a putative transcription factor shown to regulate extracellular protease secretion. However deletion of *prtT* reduced the level of secreted protease without affecting *P. digitatum* phenotypic colonization. In a different approach, treatment of inoculated fruits with the metalloprotease inhibitor 1, 10-phenanthroline inhibited the development of *P. digitatum* in citrus fruit. These findings suggest the importance of the study of metal chelation as a means to restrict micronutrient availability to pathogens.

Two papers concentrated on the interplay between *Botrytis* and tomato fruit. One analyzed the fungal factors modulating *Botrytis* pathogenicity while the other analyzed the host response to *Botrytis* colonization. The study “Characterization of the role of a non-GPCR Membrane-Bound CFEM protein in the pathogenicity and germination of *Botrytis cinerea*” is presented by Chand Arya et al. [14]. The common fungal extracellular membrane proteins (CFEM) were found to participate in various functions mediating different physiological (e.g., cell wall stability) and infection processes of fungal pathogens. In this work, the authors analyzed the role of membrane-bound and secreted CFEM-containing proteins in different aspects of *Botrytis* virulence. Functional analysis of deletion mutants, ∆CFEM-Bcin07g03260, showed a delay in conidial germination, germ tube elongation and reduced progression of a necrotic lesion on tomato leaves. Further analysis of the mutants revealed significant reduction compared with the wild type strain. Considering various functions identified for CFEM proteins in fungal virulence, the data presented illustrates a potential new role for a non-GPCR (G-protein couple receptor) membrane CFEM in pathogenic fungi to control virulence in *B. cinerea.*

The second manuscript on the interplay between *Botrytis* and tomato is “Physiological and proteomic address the active role of *Botrytis cinerea* inoculation in tomato postharvest ripening” by Tzortzakis [15]. This manuscript focuses on the host tomato response to *Botrytis* attack. This work investigated changes in quality as a direct response or systemic response of infected tomatoes to the *B. cinerea*. Pathogen infection increased lipid peroxidation, causing the production of hydrogen peroxide and oxidative stress accompanied by an increase of 6.6% to the protein yield and a downregulation of at least 39 proteins. Some of the critical proteins that were activated were antioxidant proteins, such as ascorbate peroxidase-APX1 and superoxide dismutase (SOD), increased in infected tomatoes, as these proteins are involved in reactive oxygen species detoxification. These are similar responses to those described during the quiescent stage of *Colletotrichum* [5] and in early analysis of the host response in apples infected by *Penicillium* described by Wang et al. [2]. They indicate the importance of ROS and antioxidant proteins activated in all interactions. Furthermore, in tomato infected by *Botrytis*, protein levels involved in the metabolism of carbohydrate, the pentose phosphate pathway, and terpenoid and flavonoid biosynthesis were differently affected during the colonization. They concluded that understanding the direct or indirect effects of the fungi on fruit quality and ripening through biochemical and proteome studies is important for analysis of the mechanism of *Botrytis* attack on fruits.

One manuscript analyzed the postharvest importance of pathogens in the attack of peppers. Frimpong et al. [16] described the “Identification and Toxigenic Potential of Fungi Isolated from *Capsicum* Peppers”. In this manuscript the causal fungal factors attacking *Capsicum* peppers collected from retail markets in Nigeria and Ghana were analyzed. Pepper is one of the most important vegetable crops contributing to significant foreign exchange earnings in Sub-Saharan Africa. However, the incidence of postharvest diseases has become a major constraint especially given that they are susceptible to fungal infection and subsequent contamination with mycotoxins. Forty fungal isolates belonging to 7 families, 8 genera, and 17 species were identified based on morphology, culture characteristics and DNA sequencing of the internal transcribed spacer region. *Aspergillus* spp., *Fusarium* spp. and *Colletotrichum* spp. were found to be the predominant fungal pathogens. Furthermore, the potential ability of the isolated mycotoxigenic fungi to produce some major mycotoxins was analyzed using high-performance liquid chromatography. Among the 22 isolates analyzed, 11 strains belonging to the genera of *Aspergillus*, *Fusarium* and *Penicillium* were found to produce mycotoxins, such as aflatoxin B1, gliotoxin, deoxynivalenol and citrinin. It was concluded that a better understanding of the role of fungal contaminants in pepper fruits would assist in the development of management strategies to control mycotoxin contamination.

The review manuscript that was published in the present series described a leading subject for postharvest host–pathogen interactions. The manuscript “The pattern and function of DNA methylation in fungal plant pathogens” is presented by He and co-workers [17]. Fungal plant pathogens predominantly possess four types of DNA MTase homologs, including DIM-2, DNMT1, DNMT5 and RID. Numerous studies have indicated that DNA methylation in phytopathogenic fungi mainly distributes in transposable elements, gene promoter regions and repetitive DNA sequences. As an important and heritable epigenetic modification, DNA methylation is associated with silencing of gene expression and transposon, and it is responsible for a wide range of biological phenomena in fungi. This review highlights relevant insights regarding the role of DNA methylation in the modulation of development, pathogenicity and secondary metabolism of fungal plant pathogens. While DNA methylation is reversible and dynamic, responding to environmental and physiological conditions, it may help fungal plant pathogens to avoid host defense mechanisms and is strongly involved in host–pathogen interactions. In consideration of the annual losses of fruits caused by phytopathogenic fungi and the importance of epigenetics, it is very meaningful to figure out the significant roles of DNA methylation in fruit–pathogen interactions. This is important given that Tannous et al. [18] indicated that the deletion of an epigenetic reader, *SntB*, showed an effect on the reduction of pathogenicity in *P. expansum*. However, there is still not enough evidence to clarify the direct relationship between DNA methylation and gene expression in fungi, indicating that substantial research is necessary to investigate DNA methylome of phytopathogenic fungi.

## Conclusions

Most of the published transcriptomic reviews analyzed the different responses of hosts and pathogens during pathogenicity. It is clear that susceptibility of harvested fruit to pathogen colonization is a dynamic process that is regulated by the fruit during its maturation and ripening. Disease symptoms of postharvest pathogens usually occur at later periods after fruit infection; however, early mechanisms of host and pathogen responses in conditions of non-symptom-developing pathogens are those that determine the final phenotypic response and are mostly missing in all new studies. This non-symptomatic interaction might be in part a “semi-biotrophic interplay” that involves initial penetration of the germinated hypha into different host tissues either through wounds exposing the fungus to the released cytoplasmic and vacuolar content, facilitating intimate parasitic interaction, or may be real biotrophic interactions as observed in *Colletotrichum* when the fungal ingress appears to be suspended. Why do hemi-biotrophic pathogens such as *Colletotrichum* show a quiescence interaction become necrotrophic in host fruit? Why might necrotrophic pathogens, such as *Botrytis* or *Penicillium*, also have long non-symptomatic periods following infection until they activate their necrotrophic mechanism? Is the host maturity determining this differential susceptibility comparable to ripening fruits? Which particular host factors activate this process: sugar concentrations, pH level, soluble pectin or hormone metabolism? Early work suggested that four factors modulate termination of the quiescent interaction [4]: (i) induced accessibility of cell wall substrates during ethylene evolution and softening of the climacteric fruit; (ii) reduced concentrations of preformed antifungal compounds and inducible phytoalxins; (iii) a decline in host-defense responses and (iv) conducive pH and carbon availability in the ripening host. Those factors are mainly modulated in ripening fruits; however, in future transcriptomic analysis of the dynamics of fungal and host gene expression, the fungal response to different host physiological conditions deserve to be studied. This suggests that conditions imposed by the host during fruit maturation compared to fruit ripening may be of strong importance to understanding the mechanism of non-symptom development in infected fruits. 
